# On the evolution of chromosomal regions with high gene strand bias in bacteria

**DOI:** 10.1128/mbio.00602-24

**Published:** 2024-05-16

**Authors:** Jürgen Tomasch, Karel Kopejtka, Sahana Shivaramu, Izabela Mujakić, Michal Koblížek

**Affiliations:** 1 Laboratory of Anoxygenic Phototrophs, Institute of Microbiology of the Czech Academy of Sciences, Třeboň, Czechia; Vanderbilt University School of Medicine, Nashville, Tennessee, USA

**Keywords:** genome organization, genome evolution, gene order, strand bias, Gemmatimonadota

## Abstract

On circular bacterial chromosomes, the majority of genes are coded on the leading strand. This gene strand bias (GSB) ranges from up to 85% in some Bacillota to a little more than 50% in other phyla. The factors determining the extent of the strand bias remain to be found. Here, we report that species in the phylum Gemmatimonadota share a unique chromosome architecture, distinct from neighboring phyla: in a conserved 600-kb region around the terminus of replication, almost all genes were located on the leading strands, while on the remaining part of the chromosome, the strand preference was more balanced. The high strand bias (HSB) region harbors the rRNA clusters, core, and highly expressed genes. Selective pressure for reduction of collisions with DNA replication to minimize detrimental mutations can explain the conservation of essential genes in this region. Repetitive and mobile elements are underrepresented, suggesting reduced recombination frequency by structural isolation from other parts of the chromosome. We propose that the HSB region forms a distinct chromosomal domain. Gemmatimonadota chromosomes evolved mainly by expansion through horizontal gene transfer and duplications outside of the ancient high strand bias region. In support of our hypothesis, we could further identify two Spiroplasma strains on a similar evolutionary path.

**IMPORTANCE:**

On bacterial chromosomes, a preferred location of genes on the leading strand has evolved to reduce conflicts between replication and transcription. Despite a vast body of research, the question why bacteria show large differences in their gene strand bias is still not solved. The discovery of “hybrid” chromosomes in different phyla, including Gemmatimonadota, in which a conserved high strand bias is found exclusively in a region at *ter*, points toward a role of nucleoid structure, additional to replication, in the evolution of strand preferences. A fine-grained structural analysis of the ever-increasing number of available bacterial genomes could help to better understand the forces that shape the sequential and spatial organization of the cell’s information content.

## INTRODUCTION

Most bacterial chromosomes are circular with replication starting at one origin (*ori*) and progressing in both directions toward the terminus (*ter*). Since the earliest completely sequenced genomes, it became apparent that the need for an efficient integration of replication and transcription dictates the chromosome structure (1–3). For example, highly expressed genes tend to be located closer to *ori*, taking advantage of remaining longer in a duplicated state while the DNA is copied. This is, in particular, the case for rRNA gene clusters that make up for 90% of bacterial RNA content (4). Another constraint on gene arrangement is the possibility of clashes between the replication and transcription machineries as they move with high speed along the chromosome (5). Both work with 5′−3′ directionality. The DNA polymerase copies the leading and lagging strand in the direction of and opposing the progressing replication fork, respectively. Frontal collisions between DNA and RNA polymerase complexes slow down the transcription of lagging strand genes and can cause detrimental mutations (6, 7). Indeed, a preferential encoding of genes on the leading strand—co-directional to replication—seems to be the rule for bacterial chromosomes, and it has been controversially discussed how genes prevail on the lagging strand despite the accompanying negative effects (8–13).

There are large differences in the extent of the observed gene strand bias (GSB) between bacterial phyla (14–17). In particular, on chromosomes of Bacillota (synonym Firmicutes), often more than 75% and up to 85% of all genes are encoded on the leading strand, while in most other phyla, the distribution of genes between both strands is more balanced. To date, no satisfactory explanation has been found for these differences. In few bacterial phyla, including the Bacillota, the leading and lagging strand are replicated by utilization of two distinct polymerase subunits, PolC and DnaE, respectively, while in all others, DnaE is responsible for replication of both strands (18). It has been suggested that PolC activity might be responsible for maintenance of the high strand bias (HSB) (19). However, this hypothesis was not supported when a wider range of genomes from PolC-positive and -negative phyla were analyzed (16). A recent study across the bacterial kingdom found that a higher number of inverted repeats correlates with loss of the GSB (20).

The phylum Gemmatimonadota comprises currently of only six cultured representatives. However, their ecological importance is underpinned by the discovery of hundreds of metagenome-assembled genomes (MAGs) from diverse environments (21–23). Here, we report that the chromosome of our model strain *Gemmatimonas* (*Gem.*) *phototrophica* AP64 (24) contains a region near *ter* with an exceptional high GSB comparable to the Bacillota, while in the remaining part, genes showed a rather low preference for the leading strand. This HSB region was also conserved in the other four Gemmatimonadota isolates with complete genomes. We further analyzed various PolC-positive and -negative bacterial phyla to assess the occurrence of similar chromosome architectures. We aimed to clarify how a clustered GSB can emerge and what could explain its evolutionary stability.

## RESULTS

### Quantitative assessment of the gene strand bias

**Fig 1:**
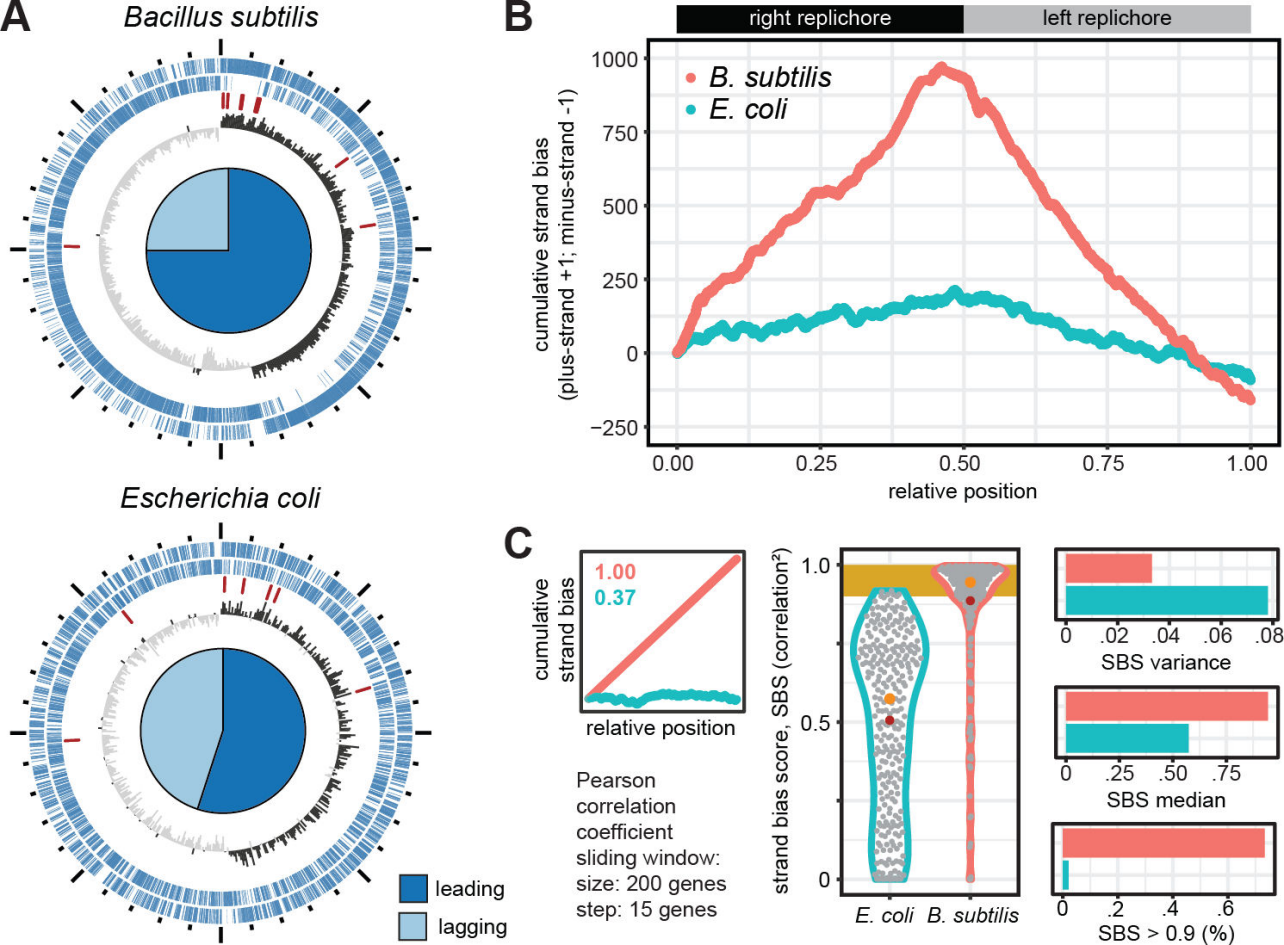
Analysis of the strand bias on circular bacterial chromosomes. (**A**) Chromosome structures of the model organisms *B. subtilis* and *E. coli*, with a high and low strand bias, respectively. Depicted, from outer to inner ring, are protein-coding genes on the plus and minus strand, rRNA genes, and the GC skew. The plots are oriented with the origin of replication at the top. The proportion of genes on the leading and lagging strand is depicted as pie chart. (**B**) Cumulative strand bias for both organisms. For each gene on the plus and minus strand, +1 and −1 are added, respectively. Counting starts on the right replichore. The position of the genes is normalized to chromosome size. (**C**) Strategy for identification of chromosomes with a high and low strand bias. The SBS is calculated as squared correlation of the cumulative strand bias with gene position, for sliding windows of 200, moving by 15 genes (left panel). Characteristics of the distribution of the SBS (middle panel) that are extracted are the median, variance, and percentage of sliding windows with SBS > 0.9 (right panel).

In order to identify HSB regions, we chose an approach developed by de Carvalho and Ferreira (25). The cumulative strand bias is calculated by moving along the chromosome of an organism and adding +1 for each gene on the plus strand and −1 for each gene on the minus strand. As exemplified for *Bacillus subtilis* and *Escherichia coli* as representatives with a high and low strand bias, respectively (Fig. 1A), this approach results in curves with a steep and flat slope, respectively, positive for the right and negative for the left replichore (Fig. 1B). Next, the cumulative GSB is correlated with the ascending positions of the respective genes for sliding windows along the chromosome. If all genes are positioned on the plus or minus strand, a correlation of 1 or −1 will be the result, respectively. For a random distribution, a value closer to zero would be expected (Fig. 1C). In the following analysis, we use the squared correlation, correcting for the direction of the bias, referred to as the strand bias score (SBS).

As we were interested in identifying larger sections of the chromosome with a conserved strand bias, we chose a sliding window size of 200, moving by 15 genes for each step. The distribution of all calculated SBS values will provide information about the overall chromosome structure. The two model organisms differ in the median and variance of the distribution of SBS values. Furthermore, the proportion of HSB regions, with an SBS higher than 0.9, is 78% for *B. subtilis* and close to zero for *E. coli* (Fig. 1C). Characterization of the SBS distribution for all analyzed genomes can be found in Table S1. For a bacterium with clustered GSB, as the Gemmatimonadota, we would expect both a high variance of the SBS and a proportion of HSB regions higher than zero.

### Gene strand bias in Gemmatimonadota and related phyla

**Fig 2:**
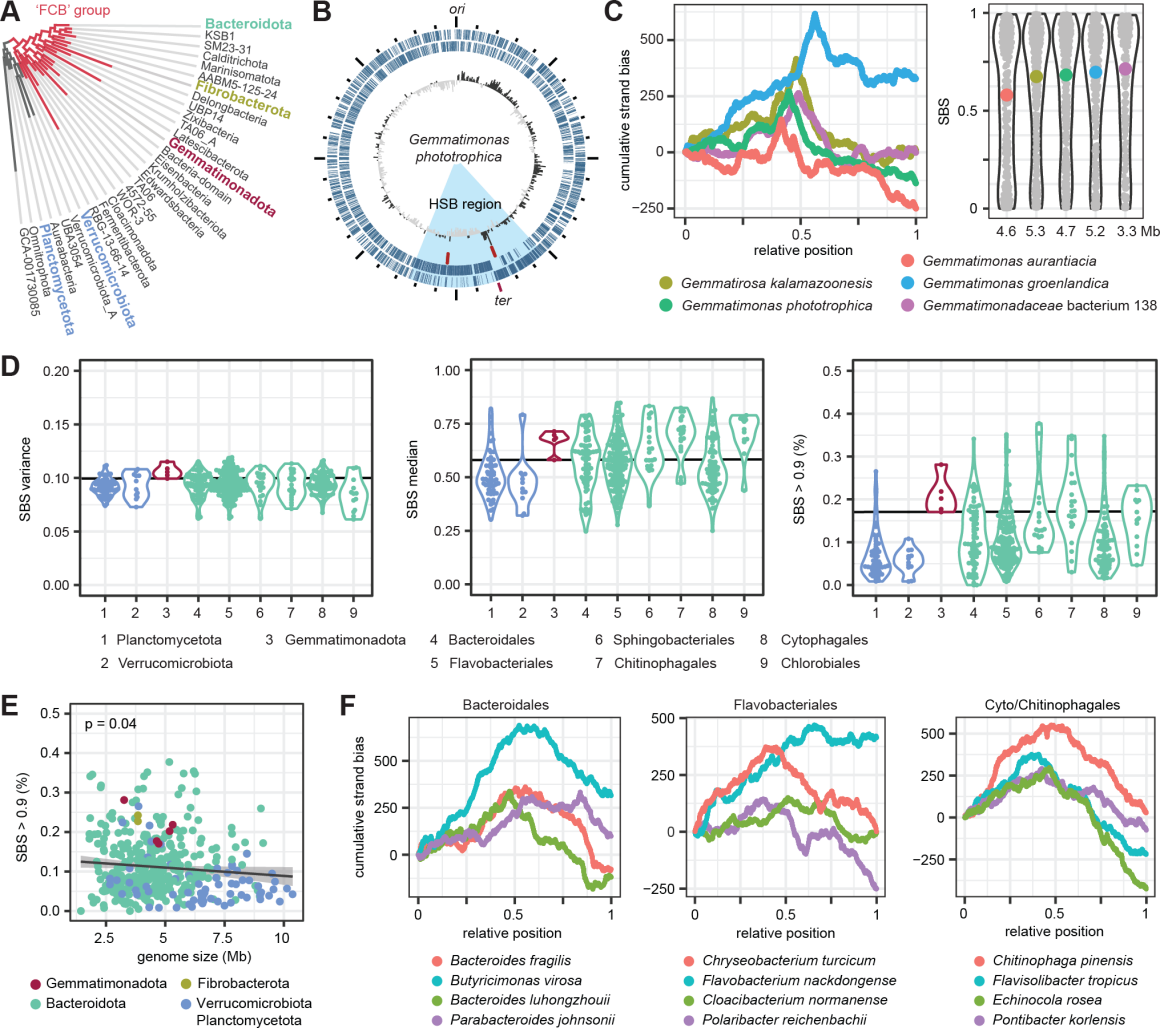
Strand bias in Gemmatimonadota and neighboring phyla. (**A**) Phylogenetic position of the Gemmatimonadota. Analyzed phyla are highlighted. Phyla without cultured representatives are shown in gray. The tree was obtained from GTDB using AnnoTree. (**B**) Chromosome of *Gem. phototrophica* AP64 centered at *ori*. The rings show genes on the plus and minus strand, the rRNA gene clusters, and the GC skew. The region with a high strand bias is highlighted in light blue. (**C**) Cumulative strand bias along the chromosome (left panel) and distribution of the SBS in sliding windows (right panel) for the five Gemmatimonadota strains with closed genomes. The median SBS is shown as colored dot. (**D**) Variance and median of the SBS in sliding windows, and the proportion of sliding windows with SBS > 0.9 in the phyla and orders, colored as in panel A. The horizontal black line marks the minimum value for Gemmatimonadota used as a cutoff for further analysis. Data for the only two Fibrobacterota strains can be found in Figure S3. (**E**) Relationship of SBS > 0.9 with genome size for all analyzed strains. The gray line indicates a fitted linear model. The *P* value of the slope is shown in the upper left corner. (**F**) Cumulative strand bias for chromosomes of selected Bacteroidota strains with SBS variance and proportion of segments with SBS > 0.9 at least as high as the cutoff.

The phylum Gemmatimonadota branches early within the so-called Fibrobacterota, Chlorobiales, and Bacteriodota (FCB) group (26). The closest earlier branching neighbors of this group with cultivated species are the Verrucomicrobiota and Planctomycetota (Fig. 2A). The closest phylum within the FCB group are the Fibrobacterota. The chromosome of our model organism *Gem. phototrophica* is characterized by a region with conserved gene order shifting from the plus to the minus strand in a region around *ter*, as derived from *ori* prediction and the GC skew (Fig. 2B). This HSB region also harbored the two rRNA gene clusters. The genome structure published in 2014 was additionally confirmed by long-read sequencing (Fig. S1). The cumulative GSB plots of all five Gemmatimonadota were characterized by a sudden steep increase and decrease around *ter*, while the remaining part of the chromosome showed regions with a weaker and also totally missing GSB (Fig. 2C). The GSB peaked at the same site as the cumulative GC skew (Fig. S2). This indicates that the switch in preferred gene directionality occurs directly at *ter*. The calculated SBS distribution was highly variable, with a considerable proportion of regions showing an SBS > 0.9.

In an attempt to trace back the evolutionary origin of this chromosomal organization, we searched for similar patterns in the neighboring phyla. The variance of the SBS was similar for all phyla but among the highest for the Gemmatimonadota, which also showed a higher proportion of HSB regions relative to the others (Fig. 2D). The chromosomes of the two earlier branching phyla, Verrucomicrobiota and Planctomycetota, were characterized by, on average, lower medians and, in particular, lower proportions of HSB regions than the FCB group strains. Within the Bacteroidota orders, the SBS medians and the number of HSB regions varied considerably. The latter ranged from almost none up to over 30% of the chromosome. Within the analyzed strains, an increasing genome size was weakly associated with a loss of conserved strand preference, in particular for the Verrucomicrobiota and Planctomycetota (Fig. 2E).

For closer inspection, we selected Bacteroidota strains with an SBS variance (0.11) and a proportion of HSB regions (0.17) at least as high as in the Gemmatimonadota (Fig. 2F). A similar arrangement along the chromosome with the characteristic but less pronounced peak at *ter* was only found for *Bacteroides luhongzhouii*, while the other strains showed a variety of patterns. For example, for *Butyricimonas virosa*, the typical V-shaped pattern of the cumulative strand bias indicated a moderate degeneration of gene orientation along the chromosome, but also showed two steep HSB stretches. In *Flavobacterium nackdongense*, only the right replichore showed a strand bias. The different slopes for the left and right replichore in *Echinocola rosea* indicate a decay of the strand preference only on the latter. One interesting case is *Fibrobacter succinogenes*, the closest relative to Gemmatimonadota among the analyzed strains. Here, a strong strand bias and stretches where it got lost are found in the *ori*- and *ter*-proximal half of the chromosome, respectively (Fig. S3).

In summary, the high variability of the strand bias between and within all analyzed phyla indicates a highly dynamic genome structure evolution. Strains with an HSB along most of the chromosome were also found, although the degree of conservation was much lower than for previously reported Bacillota. The lack of clearly shared patterns makes it difficult to follow the evolutionary path of the Gemmatimonadota HSB region at this point.

### Features of strand-biased regions compared to the rest of the Gemmatimonadota chromosomes

Next, we sought possible explanations for the emergence of the strand-biased region within the Gemmatimonadota. Therefore, we first analyzed the conservation of genes along the chromosome as a signature of genomic stability (Table S2). As only five closed genomes from cultivated strains are currently available, we determined the pan-genome of the phylum by adding 61 previously curated, high-quality MAGs (23). Next, we analyzed the distribution of transposons (Table S3) and repetitive DNA (Table S4) on the chromosome. These factors potentially contribute to genomic dynamics and expansion (27). The genomes of *Gem. groenlandica* and *Gemmatirosa* (*Gro.*) *kalamazoonesis* contained two, those of the other strains contained one incomplete phage each. (Table S5). These were not considered in the further analysis.

**Fig 3:**
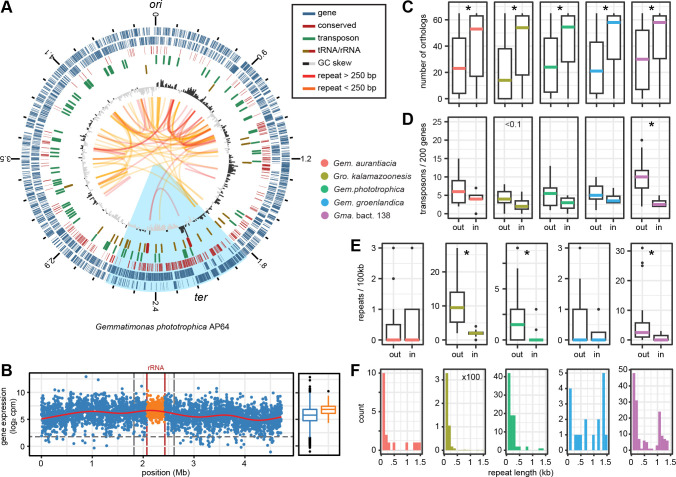
Chromosome structure of Gemmatimonadota. (**A**) Representative plot of *Gem. phototrophica*. The outer to inner rings represent genes on the plus and minus strand, genes conserved in 61 out of 65 Gemmatimonadota genomes (core genes), tRNAs and rRNAs, transposons, and the GC skew. Small (80 to 250 bp) and large (>250 bp) repetitive elements are connected by yellow and red arcs, respectively. The plot is oriented with the origin of replication at the top. (**B**) Expression of genes along the chromosome in actively growing *Gem. phototrophica* as counts per million reads normalized by gene length (rpkm). The two rRNA operons and the region between them are indicated in red and yellow, respectively. Vertical gray lines mark the borders of the HSB region. The horizontal curve represents loess-smoothed values. (**C**) Comparison of the number of orthologs per gene, (**D**) transposons per 200 genes, and (**E**) repetitive elements per 100 kb, outside and inside the strand-biased region. Asterisks indicate significant differences between inside and outside of the HSB region (Wilcoxon test, *P* value < 0.5). (**F**) Distribution of repeat length identified in five Gemmatimonadota strains. Note the individual *y*-axis scale in (**E**) and (**F**) due to the large differences in numbers of repetitive elements between the strains.

As exemplified for *Gem. phototrophica*, the HSB region differed in the studied characteristics from other parts of the chromosome (Fig. 3A). In particular, the concentration of core genes and the absence of repeats became apparent. For our model strain, we also had transcriptome data available (28) and sought to identify differences in gene activity along the chromosome (Fig. 3B). No replication-associated expression pattern was observed in accordance with the slow growth of the strain. Remarkably, the genes within the boundaries of the two rRNA operons, located inside the HSB region, showed a sharp increase in expression compared to the surrounding genes. Although this region did not contain the most highly active genes, silenced and weakly expressed genes were completely absent. This points toward a physically separated cluster of high transcriptional activity at *ter*.

In all five strains, conserved genes were significantly enriched in the HSB region (Wilcoxon test, *P* < 0.05). The median number of orthologs per gene ranged from 53 to 58 for inside, and 14 to 30 for outside this region (Fig. 3C). The position of the rRNA operons inside the HSB region was also conserved in the other strains (Fig. S4). Among others, part of the ribosomal and tRNA/rRNA-modifying genes, as well as the NADH-dehydrogenase operon, and two clusters of cell division genes were found within this region (Table S2). In *Gem. aurantiaca*, the density of core genes was visibly lower on the left replichore and the HSB region was shifted to the right replichore. The opposite arrangement was found in *Gem. groenlandica*.

The median number of transposable elements was always higher outside the HSB region (4–10 compared to 2–4 elements/200 genes). Due to the high variance of transposon distribution along the chromosomes, this difference was significant only for *Gemmatimonadota* (*Gma*.) bact. 138 (Wilcoxon test, *P* < 0.05), which had the smallest genome and the highest density of the respective genes (Fig. 3D). The five classes of transposons with the highest numbers of copies per genome were found in all five strains (Table S3). In particular, the ISArsp14 element was present in 22 to 47 copies per strain. Three transposon classes with higher copy numbers (7 to 11) were found exclusively in *Gma*. bact. 138. All strains had a smaller number of single copy transposon classes present.

The strains differed considerably in the number of repetitive elements found on the chromosome (Fig. 3E). In *Gem. aurantiaca*, only 19, and in *Gem. groenlandica*, only 20 repeats were found, with no preference to the inside or outside of the HSB region. In all other strains, the repeat density was significantly higher in the less strand-biased segments of the chromosome. *Gem. phototrophica* had 86 repeats outside and only 4 repeats located inside the HSB region; two of the latter were the rRNA gene clusters. The *Gro. kalamazoonesis* chromosome was particularly densely packed with, on average, 11 copies/100 kb of two classes of short repeats, 90 and 150 bp in size and only few longer elements (471 in total). They were also found within the HSB region but with reduced density (Fig. S4). The chromosome of *Gma*. bact. 138 showed the greatest diversity of repeats. It shared the 150 bp short sequences with *Gro. kalamazoonesis* but was also rich in 1,050 bp long repeats found in clusters exclusively outside of the HSB region (157 in total).

In summary, the concentration of core genes and the absence of repeats indicate that the Gemmatimonadota HSB region is ancient. We hypothesize that the parts of their chromosomes with a low strand preference have evolved through genome expansion, either by horizontal gene transfer (HGT) or by duplication events.

### Clustered gene strand bias in bacteria with PolC DNA polymerase subunit

**Fig 4:**
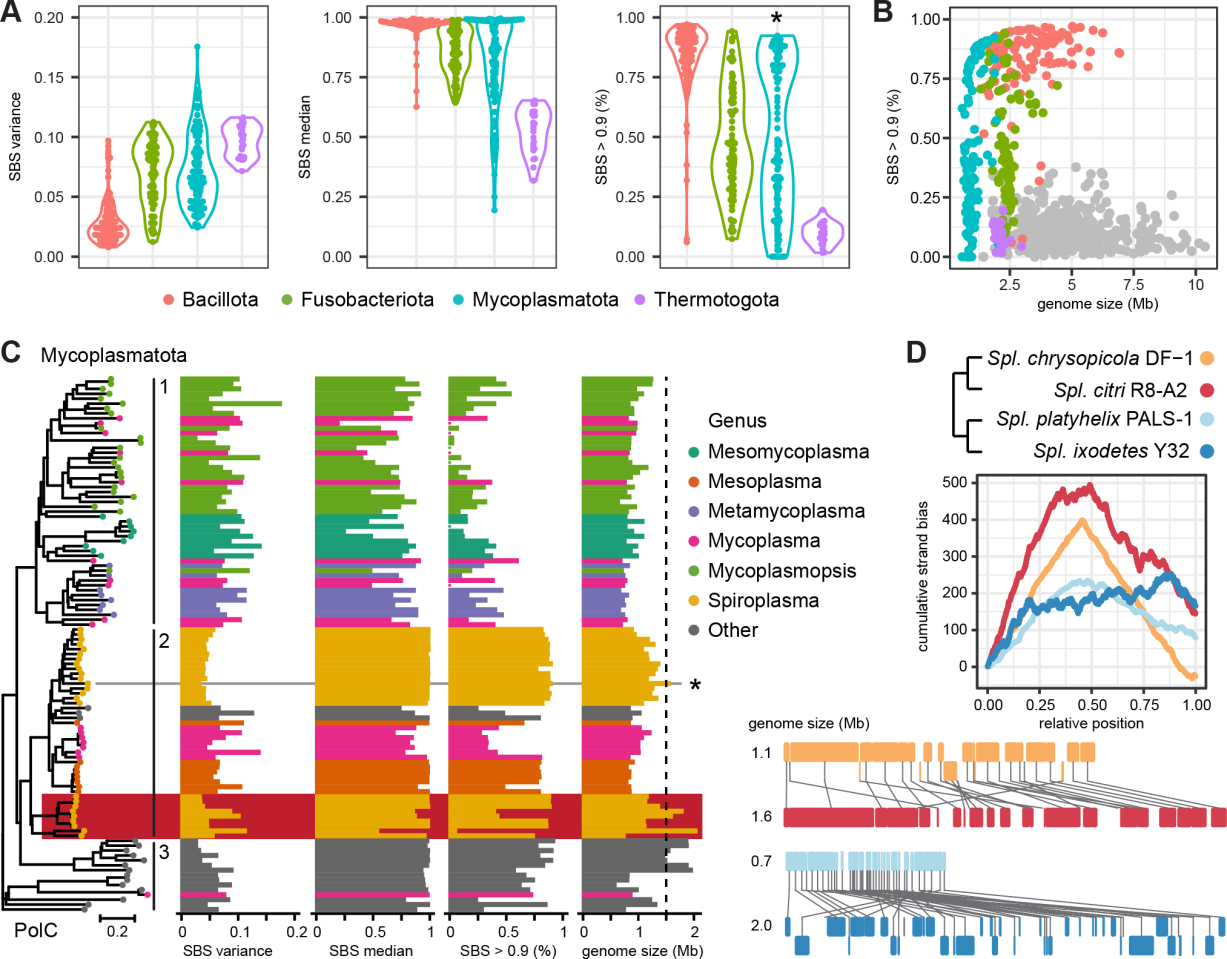
Strand bias in PolC-containing bacterial phyla. (**A**) Variance and median of SBS in sliding windows, and the proportion of sliding windows with SBS > 0.9 in the four phyla with *polC* encoded in the genome. The asterisk in the right panel indicates significant bimodality of the distribution (excess mass test, *P* value < 0.5). (**B**) Relationship of SBS > 0.9 proportion and genome size. Strains from Figure 2 are shown in gray for comparison. (**C**) Unrooted neighbor-joining tree of the Mycoplasmatota PolC protein compared to the three parameters of the chromosomal SBS distribution and genome size. Three distinct phylogenetic groups and an exceptional cluster of *Spiroplasma* strains are highlighted. A 1.5-Mb cutoff for a possible genome expansion (dashed line) was set based on the previously published *S. clarkii* case (marked with an asterisk). (**D**) Cumulative strand bias and whole chromosome alignments of two pairs of closely related *Spiroplasma* strains from the marked cluster.

To evaluate our hypothesis, we sought to identify such an expansion event in representatives of PolC-positive phyla, in which a high GSB is usually conserved along the chromosome. Besides Bacillota, forming the foundation for the proposed (and rejected) PolC dependency of the GSB (19), homologs have been identified in Fusobacteriota, Mycoplasmatota (former Tenericutes), and Thermotogota (29). In particular, the latter phylum has been previously found to lack a GSB (16). The Bacillota showed overall the lowest variation and high median of the GSB (Fig. 4A). For 116 out of 120 chromosomes, the median SBS of the sliding windows was higher than 0.9. The PolC proteins of 12 strains had large alterations of the protein structure, for example, losses of entire conserved domains (Table S6). However, the strand bias was not reduced in any of these strains (Fig. S5). The other three phyla showed significantly different patterns (Tukey’s honest significant difference [HSD], *P* value < 0.05). The Fusobacteriota had retained overall a high strand bias although to a lesser extent and more variable than the Bacillota. The Thermatogota showed the highest variance and the lowest median, and no more than 25% of the genes located in the strand-biased regions. In these aspects, their chromosomes resembled more that of PolC-negative *E. coli*. Genomes larger than 3 Mb, mostly present in Bacillota and Fusobacteriota, tended to have a higher gene strand bias (Fig. 4B).

Of all four phyla, the Mycoplasmatota showed the widest ranges of the analyzed parameters and a significant bimodality (excess mass test, *P* value < 0.05) of the HSB proportion (Fig. 4A). We investigated, therefore, if we could trace back the evolutionary loss of the GSB in specific genera within this phylum (Fig. 4C). Genome reduction in the course of adaptation to an intracellular parasitic lifestyle is a key feature of Mycoplasmatota evolution (30). Therefore, we defined a threshold of 1.5-Mb genome size for identification of potential expansion events. This value is slightly below the size of the *Spiroplasma* (*Spl.*) *clarkii* genome for which such an expansion through HGT has been documented, although without a change in the GSB (31). Based on the PolC protein alignment, three different phylogenetic groups could be distinguished (Fig. 4C). The first group, consisting mainly of *Meso*- and *Metamycoplasma* and *Mycoplasmopsis*, had an overall low strand bias. Within the second group, a high strand bias was lost in *Mycoplasma* but conserved in most *Spiro*- and *Mesoplasma* genomes. The third group of genera with a low number of representative genomes showed both low and high strand biases. In one *Spiroplasma* cluster, three exceptional large genomes showed a reduced proportion of segments with SBS > 0.9. In those, the strand bias was conserved near *ori* but to a different extent lost toward *ter*.

Comparing each to their closest relative, we observed two different evolutionary trends for the two pairs of strains (Fig. 4D and Fig. S6). Large parts of the right replichore of the *Spl. citri* chromosome (1.6 Mb) showed a strong strand bias as present on the whole chromosome of its smaller relative *Spl. chrysopola* (1.1 Mb). However, in the *ter*-proximal region and parts of the left replichore, consisting of unique DNA, genes had no strand preference. *Spl. platyhelix* showed a deterioration of the strand bias along its small chromosome (0.7 Mb) as indicated by the reduced slope of the less pointy cumulative curve. Its relative *Spl. ixodetes* (2.0 Mb) maintained gene order only close to *ori*, while no strand preference was observed for the rest of the chromosome. The deviations between these two patterns could reflect independent gains, losses, and inversions.

In summary, an HSB was only conserved in the Bacillota but lost to different degrees in the other three PolC-positive phyla. Within the Mycoplasmatota, we could trace back the evolution of *Spl. citri* in which the ancient parts of the chromosome retained higher strand preference while newly acquired parts showed a low GSB. This pattern is similar to our observations in Gemmatimonadota.

## DISCUSSION

The dramatic increase in the number of sequenced bacteria in the last two decades has led to a broad understanding of the rules governing genome evolution. Examples are the surprisingly linear correlation between chromosomal GC content and the C:N ratio of the favorite carbon sources (32), or the preference for phage integration in proximity to *ter* (33). A current study across 773 species, covering all major bacterial taxa, found conserved positions on the chromosome, in particular a bias toward *ori* and *ter*, for almost half of the identified gene families (34). The *ori*- or *ter*-proximal position of regulatory genes can be strongly conserved across an order but can also show distinct evolutionary trajectories between phyla (35, 36). Exceptional genome architectures, defying the general evolutionary trend within a phylum, have also been found. Examples include the aforementioned rare cases of genome expansion in the Mycoplasmatota (31) and the concentration of core genes at *ter* in some Rhodobacteraceae, a family usually characterized by gene conservation biased toward *ori* (37).

The questions of how the GSB has emerged, is maintained, and gets lost have been partially answered. Purifying selection can remove genes on the lagging strand if their expression interferes negatively with replication (8, 12, 38). Contrastingly, the higher mutation rate might provide a fitness benefit for genes that need to be quickly adapted, like those coding for virulence or transcription factors (10, 39). Regardless of the detrimental effects, most bacteria thrive well with a rather large fraction up to almost half of their genes oriented head-on to replication (1, 17, 40). Gene inversions, identified through a sign change in GC skew compared to the surrounding, seem to be common, although the frequency and directionality can vary between phyla (10, 40). The Bacillota remain so far the only exception with an almost universally conserved HSB, even along large chromosomes. The other PolC-positive phyla have diverged into clades with a different extent of the GSB, like the Mycoplasmatota, or lost it completely, like the Thermotogota. PolC might still be a necessary but is definitely not a sufficient prerequisite for a conservation of the strand bias along the full chromosome (16). A high inversion frequency from the leading to the lagging strand, caused by recombination of inverted repeats located on the same replichore, apparently plays an important role in the loss of the GSB (20).

The discovery of bacteria with “hybrid” chromosomes, having segments with both high and low GSB, might further help to understand the evolutionary development of strand preferences. The Gemmatimonadota chromosomes harbor a distinct 600-kb region with a pronounced GSB switching from the plus to the minus strand. This region is roughly opposite of the *ori* and peaks at the sign-change of the GC skew that does not always split the chromosome into equally sized halves. It has been shown before that the position of the *dif*-site, as a proxy for *ter*, relative to the *ori* can vary (41). We conclude that replication ends where genes switch their strand preference. Consequently, the number of genes on the leading strand and, thereby, co-directionality of replication and transcription would be maximized. Although opposing the general trend observed for other bacteria (4, 42), the position of rRNA (and core) genes at the terminus of replication is characteristic for slow-growing strains, in which gene dosage plays only a minor role (43). Expression of these genes is presumably highest during cell division when new ribosomes and other important cell components have to be synthesized. The same holds true for the *ter*-proximal cell division genes that were found to be actively transcribed during replication in other bacteria (44, 45). Co-directional transcription close to *ter* would minimize collisions with the replication machinery. This chromosomal setup would ensure that highly expressed essential genes are shielded from accumulating mutations.

**Fig 5:**
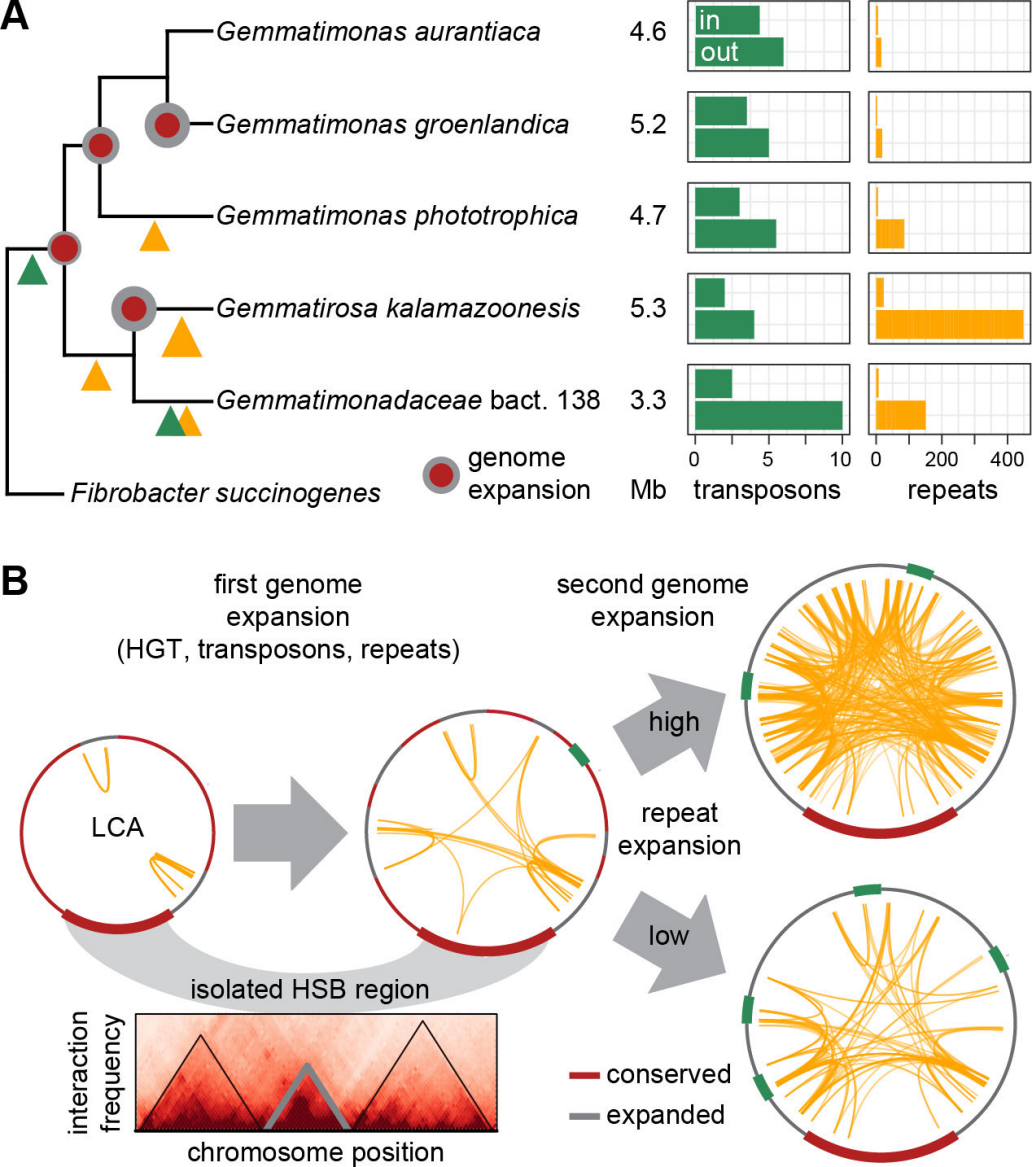
Evolution of the clustered GSB in Gemmatimonadota. (**A**) Cladogram of the analyzed strains based on 16S rRNA phylogeny. Major genome expansion events are indicated by circles at the nodes. Integration and spread of transposons and repetitive elements are indicated by triangles at the branches. The genome sizes in Mb are shown next to the strain names followed by the number of transposons per 200 genes and the total number of repeats inside and outside the GSB region. (**B**) Hypothetical scenario of Gemmatimonadota genome evolution with different timing and extent of chromosome restructuring events. Physical isolation could shield the GSB region on the folded chromosome from recombination and invasion of foreign elements. A theoretical chromosome interaction map illustrates this scenario.

How can the evolution of the Gemmatimonadota chromosome structure be explained? We suggest the following scenario (Fig. 5A): the chromosome of the last common ancestor (LCA) of the present strains was smaller than the 3.3 Mb of *Gma*. bacterium 138 and had already a *ter*-proximal-ordered gene orientation. The clustering of core genes in the HSB region might be explained by both newly acquired genes near *ori* and the loss of *ter*-proximal lagging-strand genes due to purifying selection (46). An imbalance toward gene gain by HGT would increase the chromosome size (47). Several transposon classes, present in all species, integrated into the LCA chromosome. From there, evolution of the strains took different paths. *Gma*. bact. 138 showed the least size expansion but integrated several unique transposons that have spread across the chromosome. *Gro. kalamazoonesis*, on the other hand, showed the highest number of repeats that might be partly responsible for the largest chromosome size of the analyzed strains. All three *Gemmatimonas* species had expanded genomes. Differences in the position of the HSB region relative to *ori* between *Gem. aurantiaca* and *groenlandica* indicate different replichore preferences for integration of new DNA. Repetitive elements have spread in only three out of the five analyzed strains. Thus, they are probably not the primary cause of the GSB loss in Gemmatimonadota, in contrast to the recently proposed model (20). Repeats are rather the consequence of individual duplications after the first acquisition of new genes (Fig. 5B). However, when present, they might still contribute to genome inversion and gene shuffling (27). This becomes, in particular, apparent for the repeat-rich genome of *Gro. kalamazoonesis* that shows, in contrast to the other strains, a degraded GC skew outside the HSB region, indicative for recent genomic rearrangements. Of note, the repeat density within the HSB region was always lower than outside, indicating that this it is, to some extent, shielded from the invasive spread of repetitive elements. This could partly explain the structural conservation of this region.

To understand the evolutionary stability of the HSB region, it might be important to take the three-dimensional chromosome structure into account (Fig. 5B). Facilitated by several classes of structuring proteins, the chromosome folds into a highly condensed nucleoid in the cell. In this process, subdomains are formed that differ in the density of DNA condensation and can be isolated from each other (48–50). Transcription further induces formation of boundaries between such domains (49, 51). The distinctively higher expression of genes between the two rRNA clusters could point to such a transcription-induced domain nested inside the Gemmatimonadota HSB region. A high condensation and a low interaction frequency with other DNA segments, as well as occupation by RNA polymerase complexes, would reduce the probability of recombination and also the spread of mobile elements. We propose that the Gemmatimonadota HSB region forms an isolated chromosomal domain that allows coordination of transcriptional activity with replication, but also limits the contact to other parts of the chromosome. This hypothesis is in accordance with the reduced recombination frequency between the four subdomains of the *E. coli* chromosome (52). Recently, Garmendia et al. monitored recombination-coupled repair between two non-functional copies of a marker gene in Salmonella (53). They also showed that the probability for homologous recombination can vary greatly between individual chromosomal regions and is influenced by nucleoid-structuring proteins.

Our analysis was restricted to the Gemmatimonadota, their neighbors, and PolC-positive phyla, but nevertheless documents a highly dynamic evolution of the GSB and also revealed some unique gene distribution patterns. The ever-increasing number of available complete genome sequences will help to trace back the evolution of such remarkable chromosomal structures and help to understand the forces that shape the sequential and spatial organization of the cell’s information content.

## MATERIAL AND METHODS

### Data set and tools

Genomes of the analyzed strains were obtained from the NCBI assembly database (accessed April 2023). Selection of phyla and families was guided by the GTDB database (54) and AnnoTree visualization (55). We chose only type strains with a complete genome assembly. Only the chromosomes were considered, and plasmids were discarded. Accession numbers can be found in Table S1. For all Gemmatimonadota and for additional strains selected for visualization, chromosomes were centered around the *ori*, as determined by ori-finder website (56), using the reorientCircGenomes package in R (40). Visualizations were realized using ggplot2 and ggbio (57). A complete list of the programs used in the analysis can be found in Table S7.

### Analysis of gene strand bias

The cumulative GSB was calculated as the sum of +1 for genes placed on the plus strand and −1 for genes on the minus strand. The squared correlation between the GSB and the chromosomal position, the SBS, was calculated for sliding windows of 200 kb, moving in steps of 15 kb. The sliding window size was chosen to be one-third of the Gemmatimonadota HSB region. Boundaries of the Gemmatimonadota HSB region were determined based on a stepwise increase in the SBS cutoff and manual curation. For each chromosome in the data set, the SDS variance, median, and proportion of segments with SDS > 0.9 were calculated. In addition, the mean, kurtosis, and skew of the distribution were calculated but not considered further in the analysis (Table S1). Tukey’s HSD was used to compare the distributions of these parameters between strains from different phyla and families. The excess mass test was used to identify multimodal distributions (58). Strains within the FCB group for which all three parameters were at least as high as the lowest value of the Gemmatimonadota were chosen for closer visual inspection. For comparison with the GSB, the GC skew was calculated as (G – C/G + C) from gene start to gene start.

### Analysis of genome conservation

For the Gemmatimonadota, the pan-genome was determined using proteinortho (59) using an e-value of 10^−15^ and 70% coverage as cutoffs. In addition to the five strains from NCBI, we selected 64 MAGs from a previously analyzed data set (23), with completeness >90% and contamination <5% as assessed by checkM (60). For each gene of each strain, we calculated the number of orthologs. Core genes were defined as being present in 90% of the genomes. Significant differences in the number of orthologs between chromosomal regions were identified with the Wilcoxon test. For the Mycoplasmatota, we selected the two strains with clustered strand bias and their closest relatives based on the PolC phylogeny and aligned each pair of chromosomes using mauve (61).

### Detection of mobile and repetitive elements

Transposable elements for each strain were retrieved by querying the ISfinder website (62) with the protein-coding genes and an e-value of 10^−5^ as cutoff. Only the best hit per gene was kept, and the distribution of each class of insertion sequence was determined. Repeats were identified using repseek (63), which only accepts single-entry fasta files. A custom script was used to extract the largest sequence from the assembly genomic fasta file and pipe it directly into repseek with a minimal length of 32 bp for the initial seed. Repeats overlapping more than 80 bp (half the size of the smallest detected repeat) were counted as replicated entries and discarded. Based on visual inspection of repeat length distribution, two classes were assigned, shorter or longer than 250 bp. The Wilcoxon test was used to assess the significance of differences in transposon or read distribution between inside and outside of the HSB region. Phage DNA was identified using the Phaster website (64).

### Phylogenetic analysis

PolC amino acid sequences of the Bacillota and Mycoplasmatota strains were retrieved from genomes downloaded from the NCBI GenBank (Table S1). Analyses were performed with MEGA 6.0 software (65). Sequences were aligned using the ClustalW algorithm. Ambiguously aligned regions and gaps were manually excluded from further analysis. An unrooted phylogenetic tree was inferred by using the neighbor-joining algorithm with Jones-Taylor-Thornton model and 1,000 bootstrap replicates.

### Genome sequencing and analysis

Genomic DNA of *Gem. phototrophica* AP64 was extracted using the TIANamp Genomic DNA Kit (Tiangen Biotech, Beijing, China). To obtain high-molecular-weight genomic DNA, the CTAB method was used (66). The complete genome was assembled by combining 150 bp paired-end Illumina NovaSeq 6000 reads with Oxford Nanopore long-reads as described previously (67).

## Data Availability

Genomes of all analyzed strains are publicly available at NCBI (https://www.ncbi.nlm.nih.gov/assembly). Accession numbers are provided in Table S1. Scripts are available at github (https://github.com/Juergent79/gene_strand_bias).

## SUPPLEMENTAL MATERIAL

Figure S1 mbio.00602-24-s0001.tif Long-read resequencing of *Gemmatimonas phototrophica* AP64.

Figure S2 mbio.00602-24-s0002.tif Comparison of cumulative GSB and GC skew along Gemmatimonadota chromosomes.

Figure S3 mbio.00602-24-s0003.tif Strand bias in two potentially clonal *Fibrobacter succinogenes* strains.

Figure S4 mbio.00602-24-s0004.tif Organization of Gemmatimonadota chromosomes.

Figure S5 mbio.00602-24-s0005.tif Gene strand bias in Bacillota strains with alterations in PolC.

Supplementary Figure S6 mbio.00602-24-s0006.tif Gene strand bias in *Spiroplasma* strains.

Legends mbio.00602-24-s0007.docx Legends for Figure S1–S6.

Supplemental Tables mbio.00602-24-s0008.xlsx Table S1–S7.

ASM does not own the copyrights to Supplemental Material that may be linked to, or accessed through, an article. The authors have granted ASM a non-exclusive, world-wide license to publish the Supplemental Material files. Please contact the corresponding author directly for reuse.
