# Supplementary figures and images for: On the evolution of chromosomal regions with high gene strand bias in bacteria
Source: mBio. 2024 May 16;15(6):e00602-24. doi: 10.1128/mbio.00602-24 (PMC11237797; doi:10.1128/mbio.00602-24)

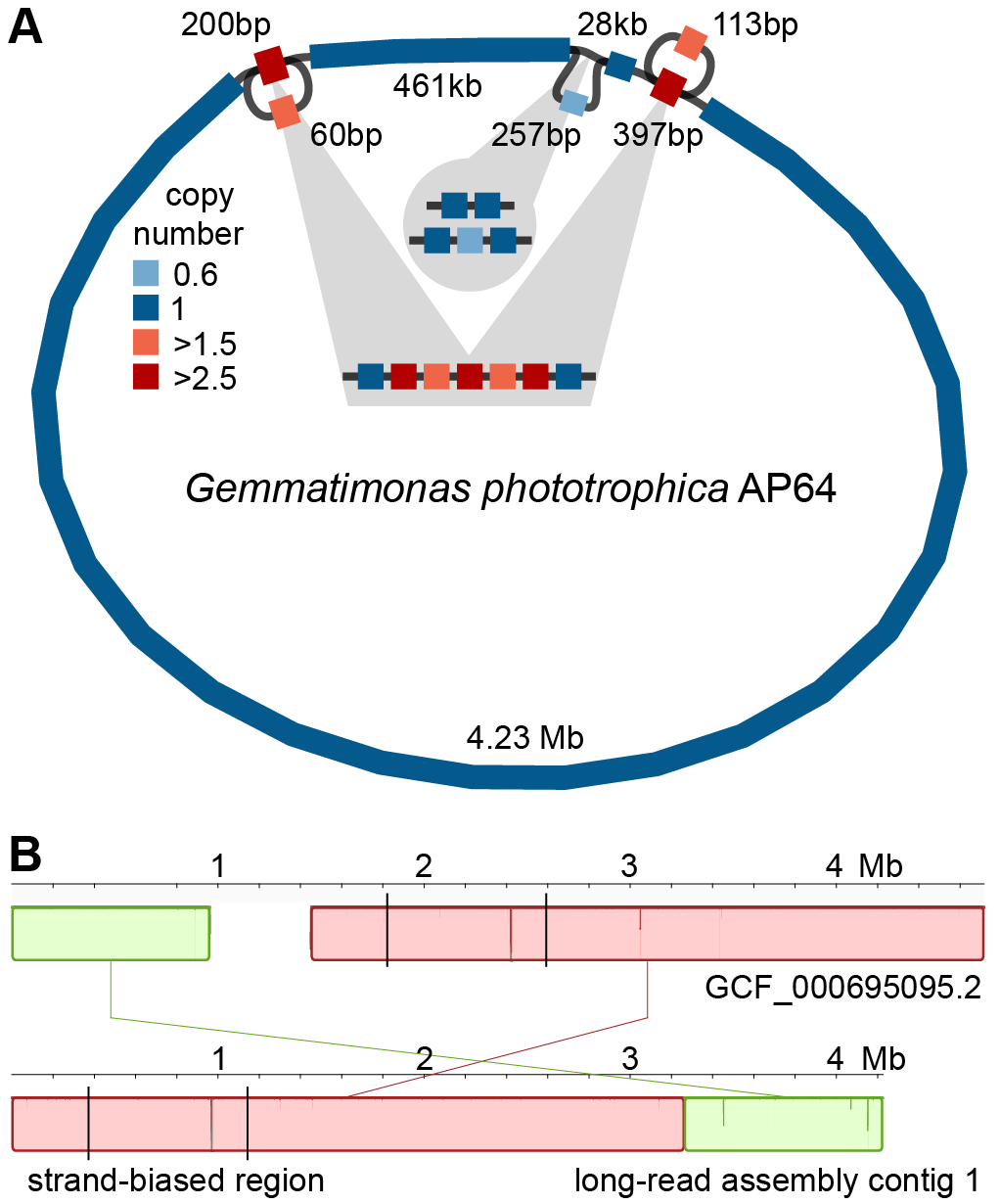

Supplement: Figure S1 — Long-read resequencing of Gemmatimonas phototrophica AP64. [file mbio.00602-24-s0001.tif]

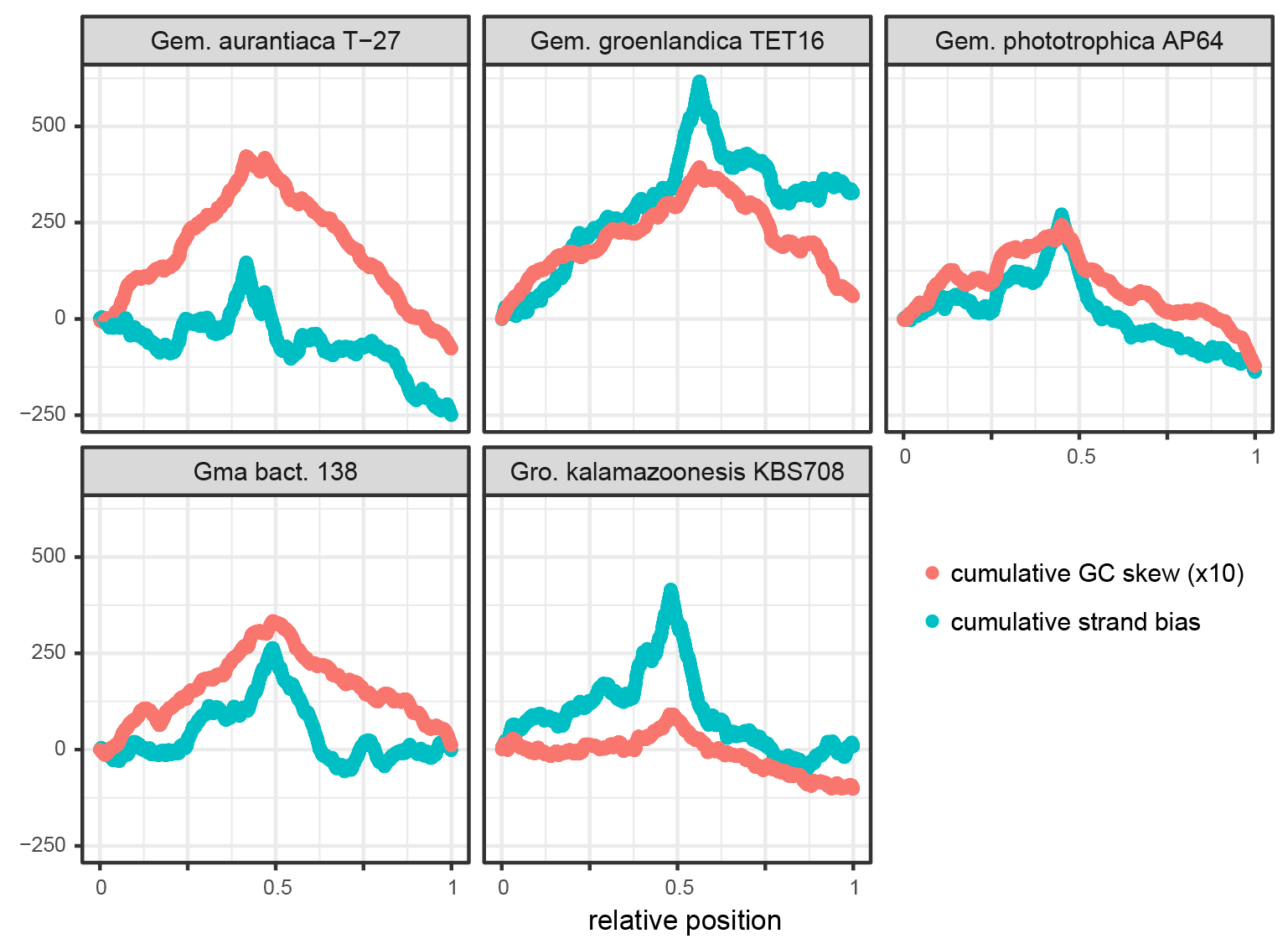

Supplement: Figure S2 — Comparison of cumulative GSB and GC skew along Gemmatimonadota chromosomes. [file mbio.00602-24-s0002.tif]

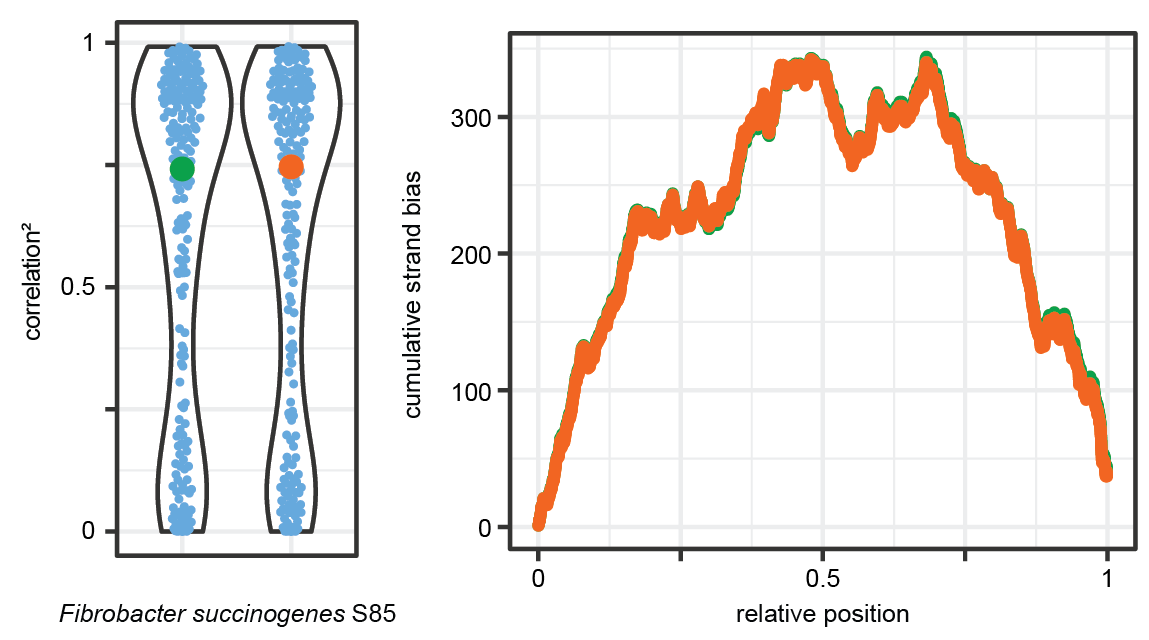

Supplement: Figure S3 — Strand bias in two potentially clonal Fibrobacter succinogenes strains. [file mbio.00602-24-s0003.tif]

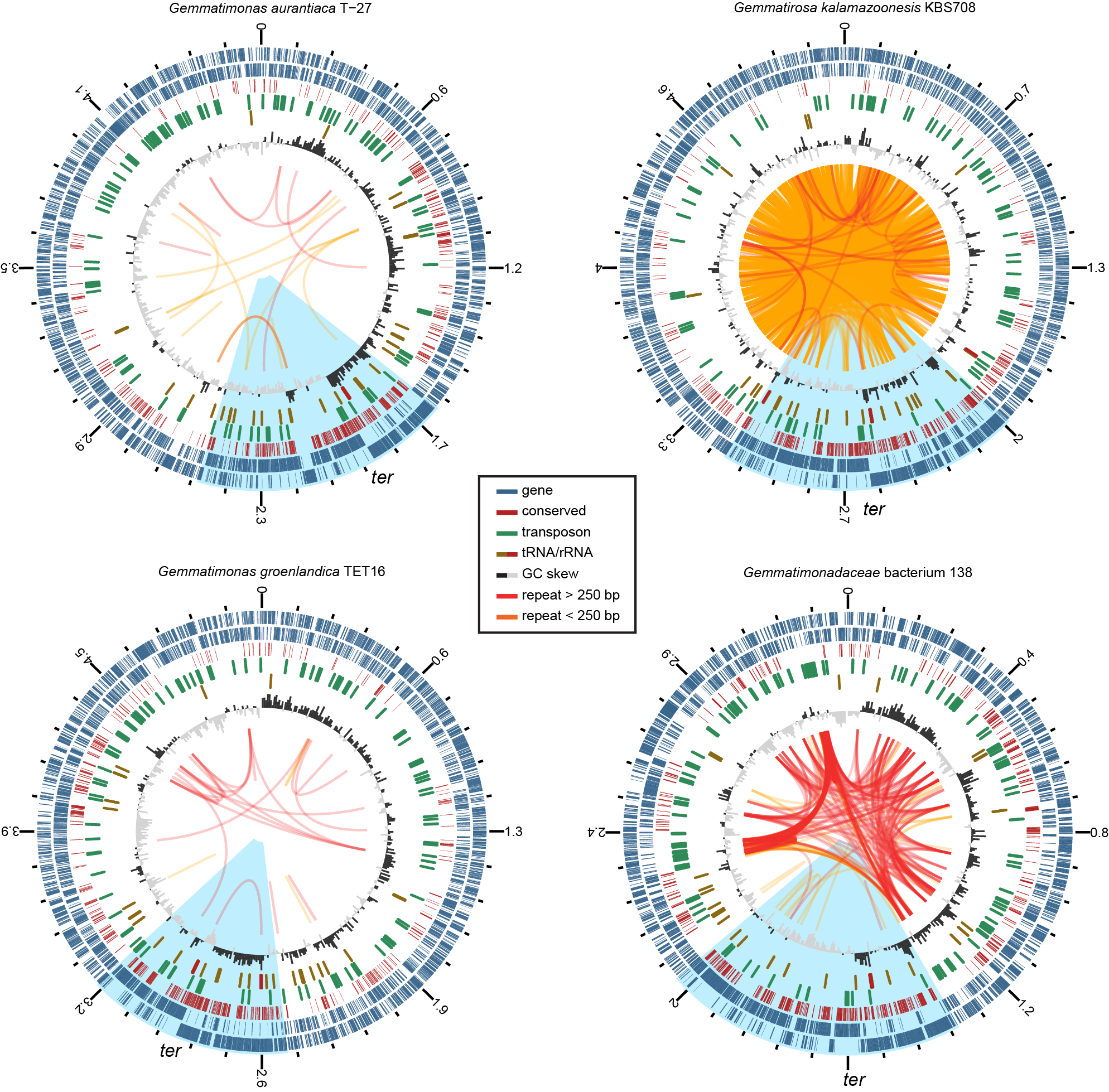

Supplement: Figure S4 — Organization of Gemmatimonadota chromosomes. [file mbio.00602-24-s0004.tif]

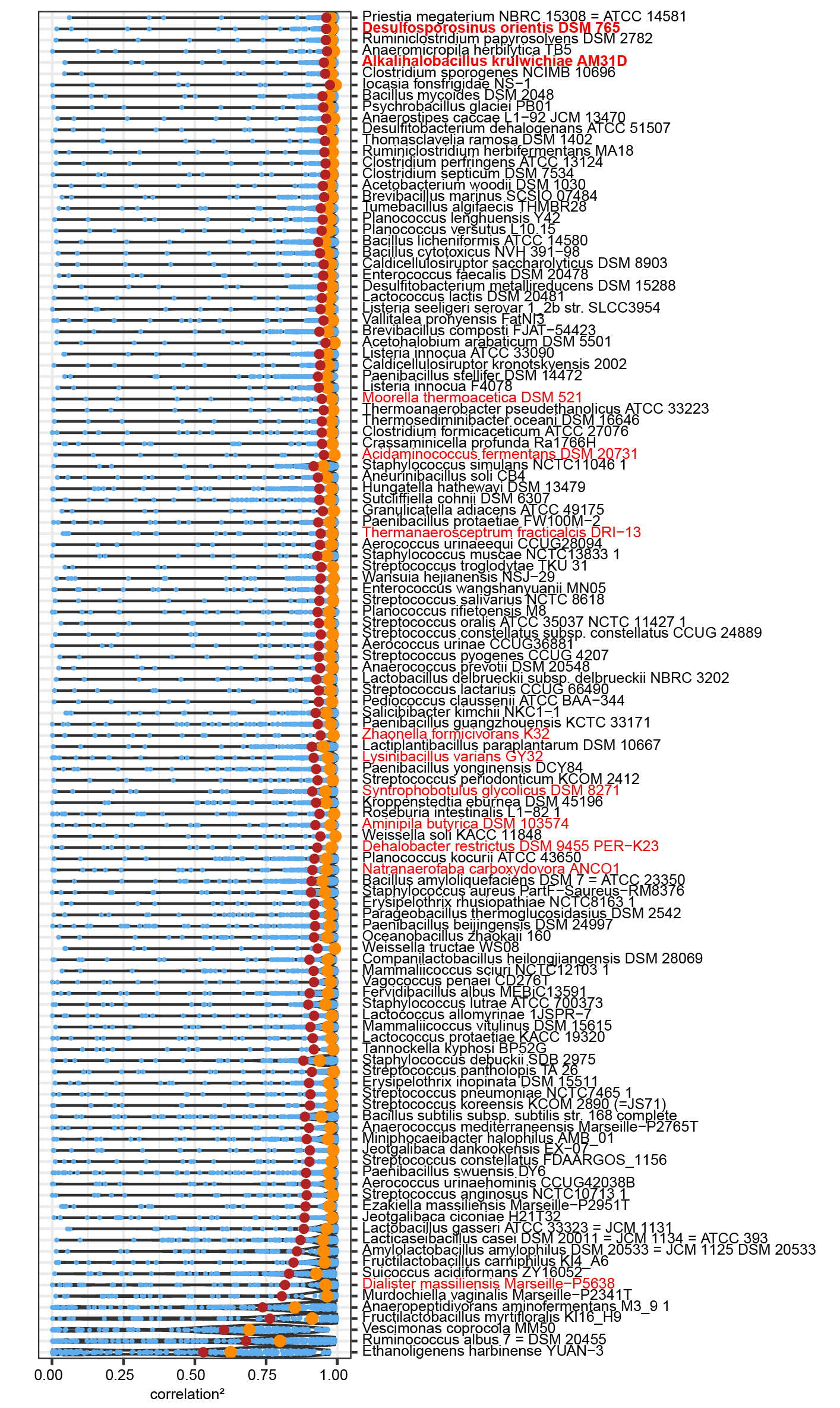

Supplement: Figure S5 — Gene strand bias in Bacillota strains with alterations in PolC. [file mbio.00602-24-s0005.tif]

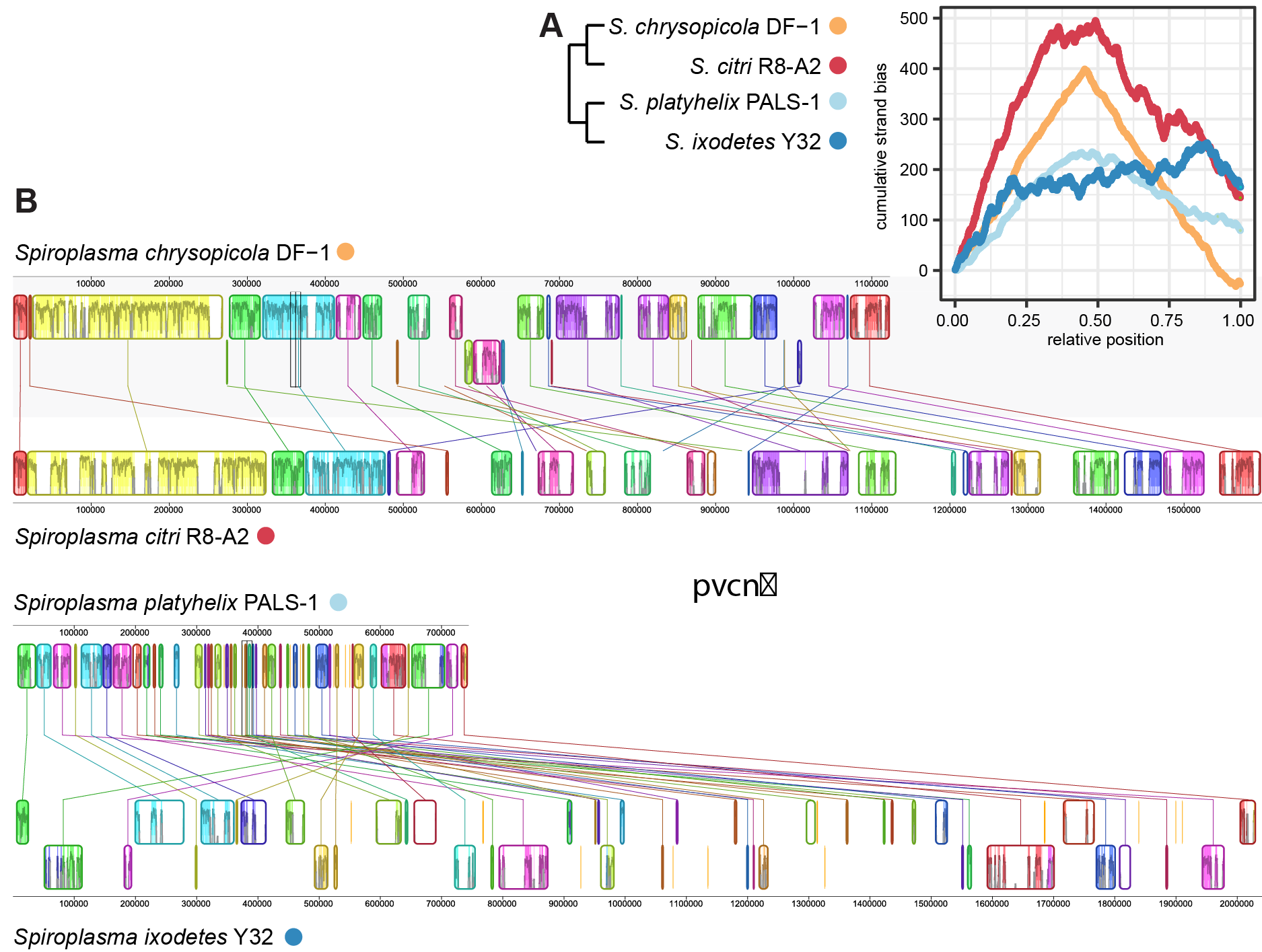

Supplement: Supplementary Figure S6 — Gene strand bias in Spiroplasma strains. [file mbio.00602-24-s0006.tif]
